# Effect of decreasing respiratory rate on the mechanical power of ventilation and lung injury biomarkers: a randomized cross-over clinical study in COVID-19 ARDS patients

**DOI:** 10.1186/s40635-025-00782-4

**Published:** 2025-07-09

**Authors:** L. Felipe Damiani, Roque Basoalto, Vanessa Oviedo, Leyla Alegria, Dagoberto Soto, M. Consuelo Bachmann, Yorschua Jalil, Cesar Santis, David Carpio, Rodrigo Ulloa, Daniel Valenzuela, Magdalena Vera, Marcus J. Schultz, Jaime Retamal, Alejandro Bruhn, Guillermo Bugedo

**Affiliations:** 1 Departamento de Medicina Intensiva, Facultad de Medicina, Pontificia Universidad Católica de Chile, Diagonal Paraguay 362, 6º Piso, P.O. Box 114D, Santiago, Chile; 2 Departamento de Kinesiología, Facultad de Medicina, Escuela de Ciencias de la Salud, Pontificia Universidad Católica de Chile, Santiago, Chile; 3 Programa de Medicina Física y Rehabilitación, Red Salud UC-CHRISTUS, 8331150 Santiago, Chile; 4 Departamento de Medicina Interna, Campus Sur. Unidad de Pacientes Críticos, Hospital Barros Luco Trudeau, Universidad de Chile, Santiago, Chile; 5 Department of Intensive Care, Academic Medical Center, University of Amsterdam, Amsterdam, The Netherlands

**Keywords:** ARDS, Invasive ventilation, Respiratory rate, Mechanical power of ventilation, MP, Ventilator-induced lung injury, VILI, Lung injury biomarkers

## Abstract

**Background:**

The respiratory rate (RR) is a key determinant of the mechanical power of ventilation (MP). The effect of reducing the RR on MP and its potential to mitigate ventilator-induced lung injury remains unclear.

**Objectives:**

To compare invasive ventilation using a lower versus a higher RR with respect to MP and plasma biomarkers of lung injury in COVID-19 ARDS patients.

**Methods:**

In a randomized cross-over clinical study in COVID-19 ARDS patients, we compared ventilation using a lower versus a higher RR in time blocks of 12 h. Patients were ventilated with tidal volumes of 6 ml/kg predicted body weight, and positive-end-expiratory pressure and fraction of inspired oxygen according to an ARDS network table. Respiratory mechanics and hemodynamics were assessed at the end of each period, and blood samples were drawn for measurements of inflammatory cytokines, epithelial and endothelial lung injury markers. In a subgroup of patients, we performed echocardiography and esophageal pressure measurements.

**Results:**

We enrolled a total of 32 patients (26 males [81%], aged 52 [44–64] years). The median respiratory rate during ventilation with a lower and a higher RR was 20 [16–22] vs. 30 [26–32] breaths/min (*p* < 0.001), associated with a lower median minute ventilation (7.3 [6.5–8.5] vs. 11.6 [10–13] L/min [*p* < 0.001]) and a lower median MP (15 [11–18] vs. 25 [21–32] J/min [*p* < 0.001]). No differences were observed in any inflammatory (IL-6, IL-8, and TNF-R1), epithelial (s-RAGE and SP-D), endothelial (Angiopoietin-2), or pro-fibrotic activity (TGF-ß) marker between high or low RR. Cardiac function by echocardiography, and respiratory mechanics using esophageal pressure measurements were also not different.

**Conclusions:**

Reducing the respiratory rate decreases mechanical power in COVID-19 ARDS patients but does not reduce plasma lung injury biomarkers levels in this cross-over study.

*Study registration* This study is registered at clinicaltrials.gov (study identifier NCT04641897)

**Supplementary Information:**

The online version contains supplementary material available at 10.1186/s40635-025-00782-4.

## Introduction

Mechanical ventilation with positive pressure is a life-sustaining therapy commonly used in the treatment of patients with acute respiratory distress syndrome (ARDS). However, it may also induce further lung injury secondary to physical mechanisms that lead to an exacerbation in the systemic inflammatory response, a phenomenon known as ventilator-induced lung injury (VILI) [1].

The mechanical power of ventilation (MP) has been proposed as a unifying concept to explain the mechanics determinants of VILI [2]. Mechanical power represents the total energy transferred from the ventilator to the respiratory system, which is derived from tidal volumes, airway pressures, and respiratory rate (RR). High MP of ventilation is associated with mortality and morbidity in patients who are critically ill and receiving invasive mechanical ventilation [3–6].

Respiratory rate (RR) is a crucial parameter when setting mechanical ventilation, since it affects CO_2_ removal and acid/base balance [7]. In ARDS, patients are typically ventilated with higher RR to compensate for the low tidal volumes (e.g., 6 ml/kg of predicted body weight [PBW]), in an attempt to maintain an adequate alveolar ventilation [8–10]. Experimental data suggest that lowering RR can mitigate VILI, as evidenced by decreased vascular permeability, pulmonary edema, perivascular hemorrhage, and inflammation, including decreased interleukin-6 (IL-6) levels [11–13].

In clinical practice, the potential benefits of reducing RR on MP may be limited by complex cardiopulmonary interactions and challenges in maintaining CO_2_ and pH homeostasis [10]. In addition, the impact of RR on VILI is difficult to quantify, which has prompted the use of cytokines, such as IL-6, as biomarkers of inflammation. Results from the LUNG SAFE and several randomized clinical trials in patients with ARDS showed that RR is a potentially modifiable factor associated with mortality [14, 15]. However, no randomized clinical trials have been specifically designed to assess the effects of RR on VILI, and most evidence comes from animal studies and observational clinical research.

Therefore, we performed a randomized cross-over clinical study to evaluate the sustained (12 h) effect of reducing respiratory rate compared to a higher respiratory rate on mechanical power and plasma biomarkers of ventilator-induced lung injury in patients with COVID-19 ARDS receiving lung-protective ventilation. We hypothesized that ventilation using a lower RR, by reducing MP, would be associated with decreased systemic levels of inflammatory biomarkers.

## Methods

### Study design and patients

We conducted a randomized cross-over clinical trial in invasively ventilated patients with moderate to severe ARDS caused by COVID-19 in the intensive care units of 2 hospitals in Santiago, Chile, between March 2020 and February 2022. The study protocol was approved by the Research Ethics Committee of the Pontificia Universidad Católica de Chile (ID: 180813016) and registered with ClinicalTrials.gov (NCT04641897).

Patients were eligible if (1) receiving invasive ventilation, and (2) within the first 48 h of start of ventilation, for (3) COVID-19 ARDS according to Berlin definition [16]. Exclusion criteria were patients with mild ARDS, previous chronic respiratory disease, hypercapnic or catastrophic respiratory failure, severe metabolic acidosis, hemodynamic instability, and limitation of therapeutic effort (see supplementary material for a full list of inclusion and exclusion criteria).

### Study interventions

During two periods, each lasting 12 h, patients were ventilated with two different RR strategies. Using a single sequence of random assignments generated by R project for Statistical Computing, patients were randomized to receive one of the following sequences (Figure S1): Sequence A, which received a lower RR during Period 1 followed by higher RR during Period 2, or Sequence B, which received higher RR during Period 1 followed by lower RR during Period 2.

Lower and higher RR targets were individualized based on a designed nomogram (Figure S2) as previously described [17]. In brief, the nomogram considers baseline RR, pH and PaCO_2_, and set a range of RR where pH and PaCO_2_ can be maintained within safety limits (pH 7.20 to 7.45 and PaCO_2_ 35 to 60 mmHg). A minimum difference of 8 breaths/min between the high and low RR periods was considered relevant and clinically appropriate. Once defined the target RR, this was decreased or increased in steps of 4 breaths, each lasting for 30 to 45 min, until the target RR was obtained. Arterial-blood gases (ABGs) were measured at 2 h and repeated at 6 and 12 h. At any time, changes in RR could be made to keep PaCO_2_ and pH values within safety limits. This procedure was repeated at the beginning of every period (e.g., baseline and 12 h).

Ventilatory parameters, other than RR, were maintained throughout the protocol, including I:E ratio, volume-controlled mode with a tidal volume (Vt) 6 ml/kg IBW, and PEEP and FiO_2_ adjusted according to the ARDSNet table [8] to maintain oxygen saturation (SpO_2_) 88–95% and PaO_2_ between 55 and 80 mmHg. If the PaO_2_:FiO_2_ ratio was less than 150 mmHg, patients were positioned in a prone position before the study inclusion and maintained throughout the study protocol [18]. All patients received deep sedation and neuromuscular blockade during the study period [19].

### Study measurements

The following demographics were collected: age, gender, body mass index (BMI), APACHE II at admission, SOFA score, use of steroids and comorbidities.

The main outcome of the present study was IL-6 levels in plasma, a pro-inflammatory cytokine which has been extensively studied in ARDS patients as a marker of VILI [8, 20, 21]. Secondary outcomes included the level of energy applied to the lungs (i.e., the mechanical power), other lung injury biomarkers, gas exchange, respiratory mechanics, and hemodynamic variables.

More advanced physiological measurements were performed in a subgroup of patients, including transpulmonary pressures, extravascular lung water index (EVLWI) and the presence of acute dysfunction of the right ventricle (acute cor pulmonale) by echocardiography (see details on the online data supplement).

### VILI biomarkers

Plasma levels of inflammatory cytokines and mediators (IL-6, IL-8, and TNF-α receptor [TNF-RI]), markers of epithelial and endothelial lung injury, including surfactant protein (SP-D), soluble receptor of advanced glycation end-products (RAGE) and angiopoietin-2, and early pro-fibrotic activity (TGF-β), were quantified as previously described by commercially available ELISA kits (R&D Systems, USA) [22, 23] at baseline and at the end of each period (12 and 24 h) to compare the effects of high vs. low RR.

### Respiratory variables

Ventilatory variables and respiratory mechanics were measured at baseline, 12 and 24 h. Static respiratory system compliance and driving pressure were calculated as previously described [24]. MP was estimated based on previous description [4].

An esophageal catheter (Cooper Surgical, Trumbull, CT) was placed to measure esophageal pressure (Pes) and estimate respiratory mechanics and transpulmonary pressure (P_L_ = Paw–Pes). Flow, airway pressure (Paw) and Pes were collected the last 5 min at different study timepoints on a personal computer-based data acquisition system (Hans Rudolph 3700; Kansas City, MO). Waveforms from Pes, Paw, and PL were analyzed with AcqKnowledge software (Biopac Systems, Santa Barbara, CA).

### Hemodynamic variables

At baseline and at the end of each period, we collected ABGs, heart rate and arterial-blood pressure. A femoral or brachial arterial catheter with the PICCO system (PV2015L20, Pulsion, Munich, Germany) was inserted for cardiac output and extravascular lung water measurements in a subgroup of patients. Transthoracic echocardiography examinations (Mindray Bio-Medical Electronics Co., Shenzhen. China) were performed for cardiac function assessment at the end of each study period (Low and High RR).

### Sample size calculation

*According* to previous clinical studies [8, 20, 21, 25], we expected a mean (SD) difference of 20% (27%) on IL-6 values between RR interventions. Therefore, we calculated the sample size for a power of 0.8 and a two-sided alfa-error of 0.05, obtaining 31 patients for a cross-over study.

### Statistical analysis

Data were expressed as median and 25–75% interquartile range (IQR). The paired Wilcoxon signed-rank test was used to compare ventilatory, hemodynamic and lung injury markers between high and low RR interventions regardless of the study sequence. In a sub analysis, patients were divided according to the median respiratory system compliance, median baseline level of IL-6, and ARDS severity (severe or moderate) at ICU admission to compare the primary outcome (i.e., IL-6) between RR interventions. All statistical analyses were performed by R software version 3.3.2 (R project for Statistical Computing). Statistical tests were carried out with a significance level of 0.05.

## Results

### Patients

Thirty-two patients were enrolled, and their main characteristics are reported in Table 1. Twenty-six were men (81%), with a median age of 52 [44–64] years and BMI of 31.6 [29.3–35.4] kg/m^2^. APACHE II and PaO2:FiO2 ratio at ICU admission were 12 [11–15] and 92 [77–122], respectively. Main comorbidities were hypertension (28%) and diabetes mellitus (19%). Nineteen patients (59%) had severe ARDS and 25 (78%) remained in prone position throughout the study. The median duration of MV before randomization was 36 [24–40] h. Sixteen patients were allocated to each sequence without differences between groups at baseline. All patients successfully completed the study protocol. Regarding the follow-up of patients after the study protocol, total mechanical ventilation duration, and ICU length of stay were 12 [9–29] and 16 [13–33] days, respectively, and hospital mortality was 19%.

**Table 1:** Demographics, ventilatory and hemodynamics characteristics in all patients based on randomization arm

Variable	Total (*n* = 32)	High RR–low RR (*n* = 16)	Low RR–high RR (*n* = 16)
Age, years	52 [44–64]	50 [44–55]	56 [39–69]
Sex, no. (%)			
Female	6 (19)	4 (25)	2 (13)
Male	26 (81)	12 (75)	14(88)
Body mass index, kg/m^2^	31.6 [29.3–35.4]	32.2 [30.4–36.7]	30.1 [29.0–35.2]
SOFA	8.0 [6.7–9.0]	8.0 [6.7–9.5]	8.0 [6.7–9.0]
APACHE II	12 [11–15]	11 [9–17]	13 [11–14]
Comorbidities, no. (%)			
Hypertension	9 (28)	5 (31)	4 (25)
COPD	1 (3)	0 (0)	1 (6)
Diabetes mellitus	6 (19)	4 (25)	2 (13)
Coronary artery disease	3 (9)	1 (6)	2 (13)
Other	4 (13)	1 (6)	3 (19)
Prone position, no. (%)	25 (78)	11 (69)	14 (88)
Use of corticosteroids, n (%)	18 (60)	9 (60)	9 (60)
Ventilatory and arterial-blood gases*			
Tidal volume (mL/kg PBW)	6.1 [5.9–6.6]	6.2 [5.9–6.7]	6.1 [5.8–6.3]
Respiratory rate (bpm)	26 [24–28]	24 [23–26]	27 [24–28]
PEEP (cmH_2_O)	10 [8–12]	10 [8–11]	10 [8–13]
Cst (mL/cmH_2_O)	37 [30–42]	35 [32–43]	37 [28–42]
Mechanical Power, J/min	20.9 [18.1–24.3]	19 [18–21.6]	22.4 [19.6–25]
PaO_2_:FiO_2_ (mm Hg)	150 [116–198]	146 [104–198]	157 [130–201]
PaCO_2_ (mm Hg)	44 [38–47]	46.5 [38.0–49.3]	42.0 [37.5–45.6]
Arterial-blood pH	7.40 [7.34–7.42]	7.36 [7.32–7.41]	7.41 [7.38–7.43]
Heart rate (bpm)	71 [63–84]	78 [64–99]	68 [59–77]
Mean arterial pressure (mmHg)	80 [72–83]	81 [75–83]	79 [72–83]

Data expressed in median [p25–p75%], and no. (%)

^*^Ventilatory variables and arterial-blood gases were collected at baseline

*COPD* Chronic obstructive pulmonary disease, *ARDS* Acute respiratory distress syndrome, *PEEP* positive-end-expiratory pressure, *BMI* Body mass index

^*^p value for comparison between high RR–low RR and low RR–high RR using Wilcoxon signed-rank test, chi-squared or Fisher's exact test

### Changes in respiratory rate

**Fig. 1:**
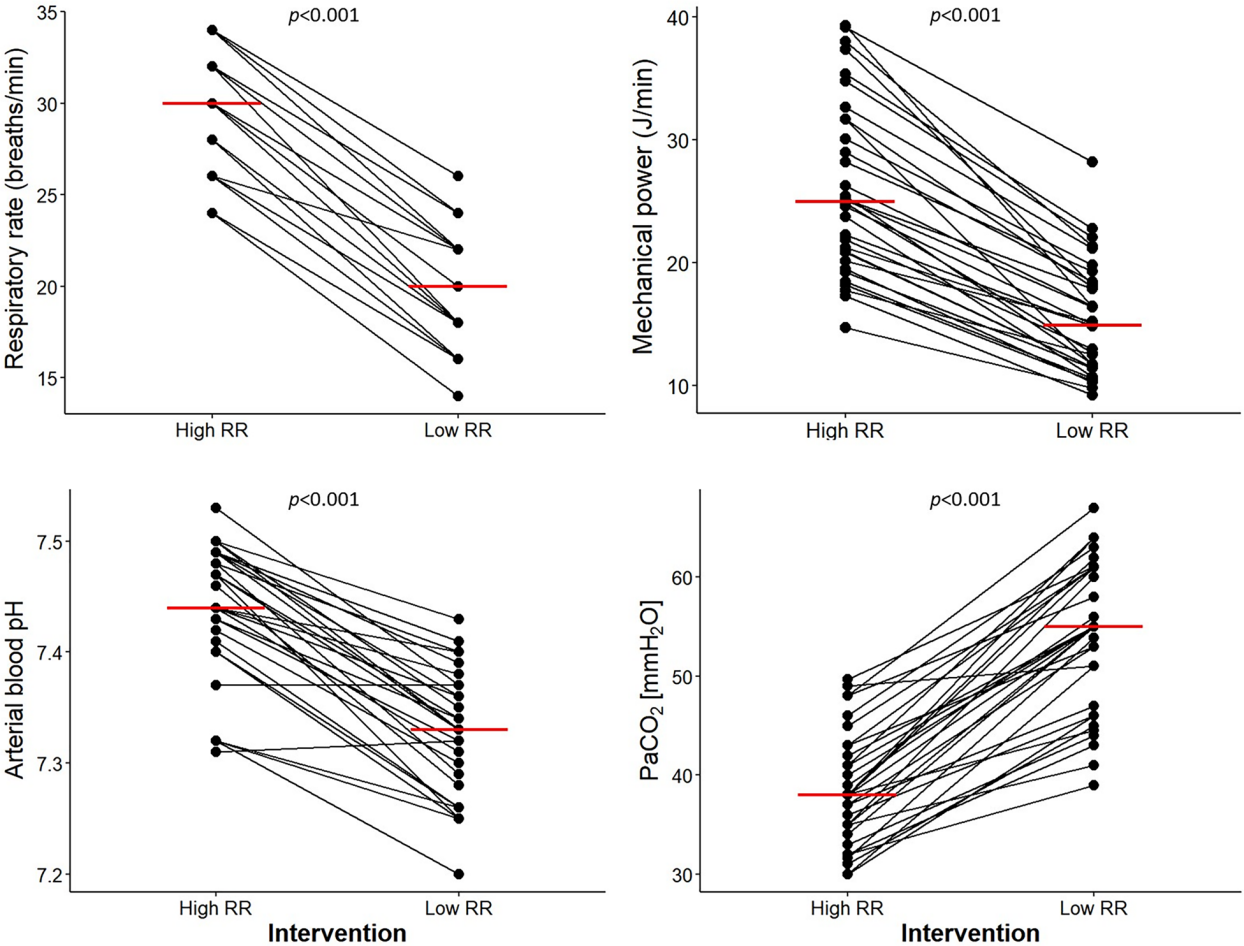
Respiratory rate, PaCO_2_, pH, and mechanical power of ventilation according to high and low respiratory rate interventions. Compared with the low respiratory rate, high respiratory rate significantly increased mechanical power of ventilation. High respiratory rate yielded higher arterial-blood pH and lower PaCO_2_ than low respiratory rate**.** Individual values and medians (horizontal red lines) are displayed in all panels

The target RR was achieved within 2 h in most patients, and all patients finished the 24-h protocol. The median [IQR] difference in RR intervention between ventilation using a lower and a higher RR was 10 [10] breaths/min. The evolution of RR values for each patient throughout the study protocol is shown in supplementary Figure S3. As compared to ventilation using a higher RR, ventilation using a lower RR resulted in a lower median minute ventilation (7.3 [6.5–8.5] vs. 11.6 [10–13] L/min [*p* < 0.001]), and less median MP 15 [11–18] vs. 25 [21–32] J/min [*p* < 0.001]), respectively. PaCO_2_ was higher and arterial pH was lower during ventilation with a lower RR (Table 2). The changes in individual values for RR, MP, pH and PaCO_2_ between high and low RR are presented in Fig. 1**.**

**Table 2:** Ventilator settings, respiratory system mechanics, hemodynamic and results of arterial–blood gas measurements according to high or low respiratory rate

Variable	High RR (*n* = 32)	Low RR (*n* = 32)	*p* value*
Ventilator settings			
Tidal volume (mL)	400 [364–429]	400 [361–441]	0.871
Tidal volume (mL/kg PBW)	6.0 [5.8–6.4]	6.1 [5.9–6.3]	0.999
Respiratory rate (bpm)	30 [26–32]	20 [16–22]	** < 0.001**
PEEP set (cmH_2_O)	10 [8–12]	10 [8–12]	0.683
PEEP total (cmH_2_O)	10 [8–12]	10 [8–12]	0.814
Inspiratory time, sec	0.67 [0.60–0.71]	0.99 [0.90–1.13]	** < 0.001**
Flow, L/min	36 [32–39]	23 [21–28]	** < 0.001**
Minute ventilation (L /min)	11.6 [10–13]	7.3 [6.5–8.5]	** < 0.001**
Respiratory system mechanics			
Pplat (cmH_2_O)	22 [19–24]	21 [20–24]	0.74
Ppeak (cmH_2_O)	28 [26–30]	26 [23–28]	**0.009**
Pmean (cmH_2_O)	14 [14–17]	14 [12–17]	0.322
Cst (mL/cmH_2_O)	38 [30–41]	37 [32–45]	0.361
Mechanical Power, J/min	25 [21–32]	15 [11–18]	** < 0.001**
Mechanical Power by IBW, J/min/kg	0.37 [0.33–0.45]	0.23 [0.18–0.28]	** < 0.001**
Driving pressure (cmH_2_O)	12 [10–12]	11 [10–12]	0.139
Ventilatory ratio	1.7 [1.6–2]	1.6 [1.4–1.9]	0.082
Hemodynamic and arterial-blood gas			
Heart rate (bpm)	63 [55–80]	71 [66–80]	0.064
Systolic arterial pressure (mmHg)	113 [104–119]	111 [102–126]	0.929
Diastolic arterial pressure (mmHg)	67 [59–72]	63 [58–73]	0.563
Mean arterial pressure (mmHg)	85 [76–91]	80 [74–91]	0.623
Capillary refill time, sec	2 [2–2]	2 [2–2]	0.789
Central venous pressure (mmHg)	10.5 [6.8–13]	11 [9.0–15]	0.337
PaO_2_:FiO_2_ (mm Hg)	182 [152–206]	193 [146–231]	0.617
PaCO_2_ (mm Hg)	38 [35–42]	55 [47–61]	** < 0.001**
Arterial-blood pH	7.44 [7.42–7.48]	7.33 [7.29–7.37]	** < 0.001**

Data are presented as median [p25–p75%]

*PBW* predicted body weight, *PEEP* positive-end-expiratory pressure, *Pplat* end inspiratory plateau airway pressure, *Pmax* maximal inspiratory airway pressure, *Pmean* mean airway pressure, *Cst* static compliance of the respiratory system, *FiO2* fraction of inspired oxygen, *PaO2* partial pressure of arterial oxygen, *PaCO2* partial pressure of arterial carbon dioxide, *PaO2:FiO2* ratio of the partial pressure of arterial oxygen to the fraction of inspired oxygen

Driving pressure was calculated as Pplat minus PEEP. * *p* value for high RR v/s low RR comparison using Wilcoxon signed-rank test

### Lung injury biomarkers

**Fig. 2:**
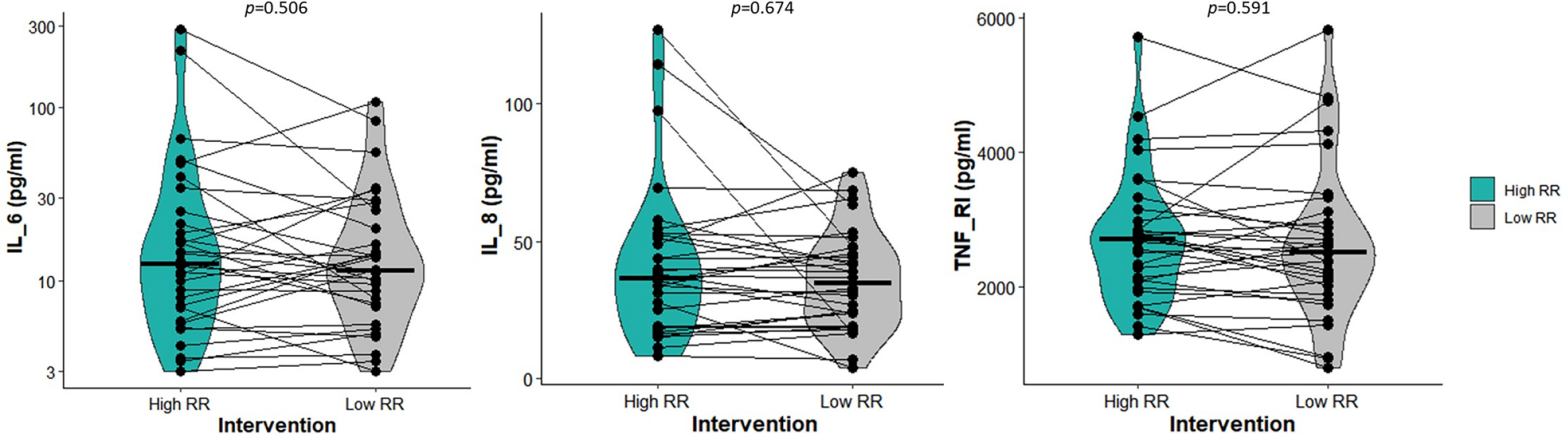
Plasma levels of inflammatory cytokines according to high or low respiratory rate interventions and study period. No significant difference was observed in IL-6, IL-8 and TNF-α receptor 1 between high (green) and low (gray) respiratory rates. Individual values, medians (horizontal lines) and violin plots are displayed in all panels. IL-6 is presented in log scale

**Fig. 3:**
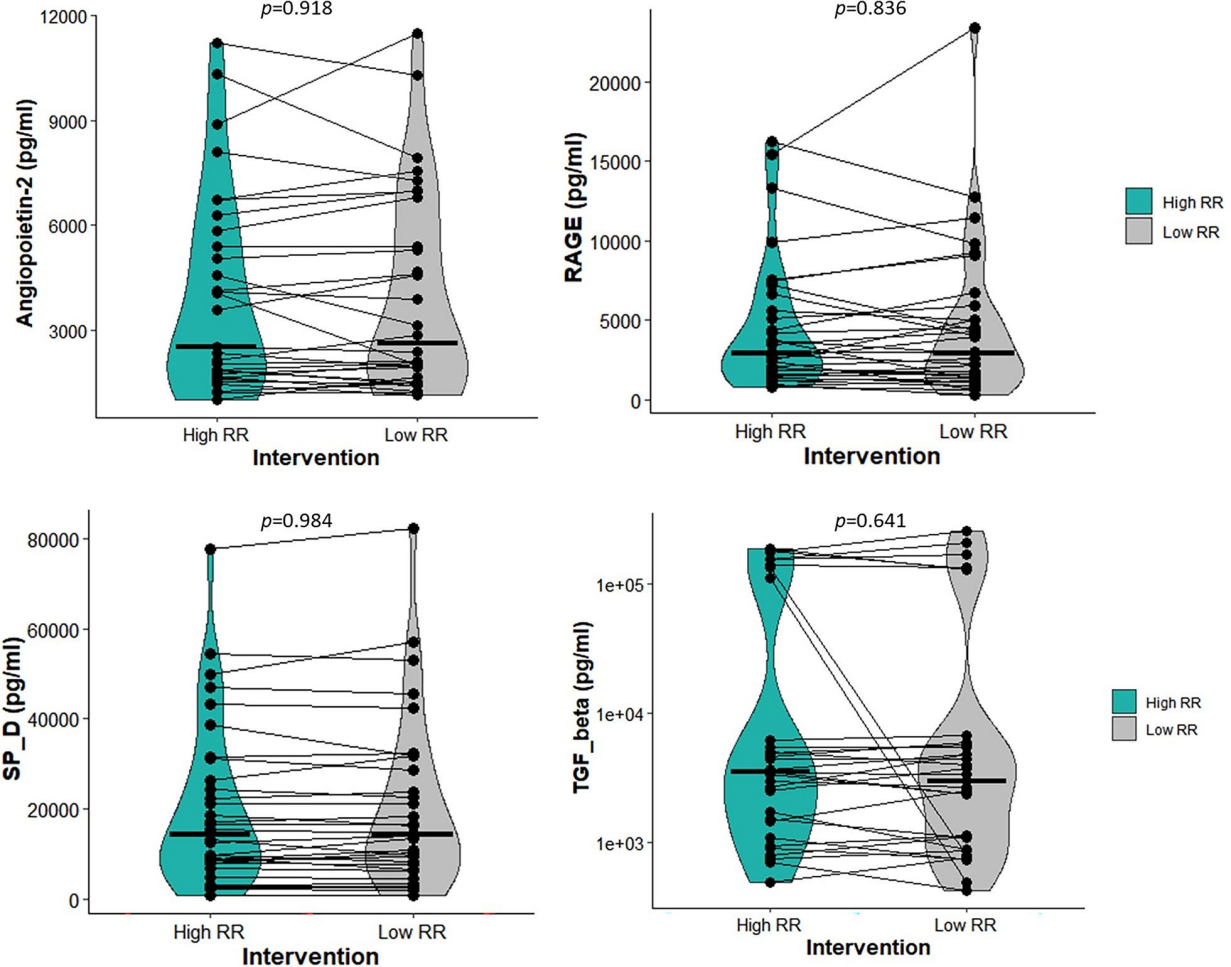
Epithelial, endothelial and fibroproliferative plasma levels of lung injury biomarkers according to high or low respiratory rate. No significant difference was observed in angiopoietin-2, surfactant protein D (SP_D), soluble receptor of advanced glycation end-products (RAGE) and transforming growth factor (TGF-β) between high (green) and low (gray) respiratory rates. Individual values, medians (horizontal lines) and violin plots are displayed in all panels. TGF-β is presented in log scale

No significant differences were observed in median plasma IL-6 levels (14 [7–22] vs. 11 [7–22] pg/ml, *p* = 0.506) nor in other inflammatory biomarkers (Fig. 2). Biomarkers of epithelial and endothelial injury (s-RAGE, SP-D, and Angiopoietin-2), and the marker of pro-fibrotic activity, TGF-β, did not differ between the periods with high and low RR (Fig. 3). The specific values for all cytokines at baseline and at the high and low RR interventions are shown in supplementary Table S1. In addition, no significant changes were observed for any lung injury biomarker when comparing the first and the second study period, irrespective of the RR intervention (supplementary Table S2).

Plasma IL-6 levels were also not different when comparing subgroups, based on ARDS severity at ICU admission, median compliance of the respiratory system (above or below the median value of 36.6 ml/cmH_2_O), or based on baseline levels of IL-6 levels (above or below the median value of 12.7 pg/ml) (supplementary Figure S4). In addition, no significant differences were observed in any cytokines or lung injury biomarkers when evaluating the effect of RR stratified by ARDS severity (Supplementary Figures S5 and S6).

### Hemodynamic variables

The heart rate and systolic, diastolic, and mean arterial pressure did not vary between high and low RR interventions (Table 2). Transthoracic echocardiography was performed in 12 patients at the end of both cross-over periods showing no differences in cardiac output, left or right ventricular function and diastolic function parameters when comparing high vs. low RR (supplementary Table S3). Extravascular lung water (EVLW) measurements were also not different.

### Respiratory mechanics and transpulmonary pressure

**Fig. 4:**
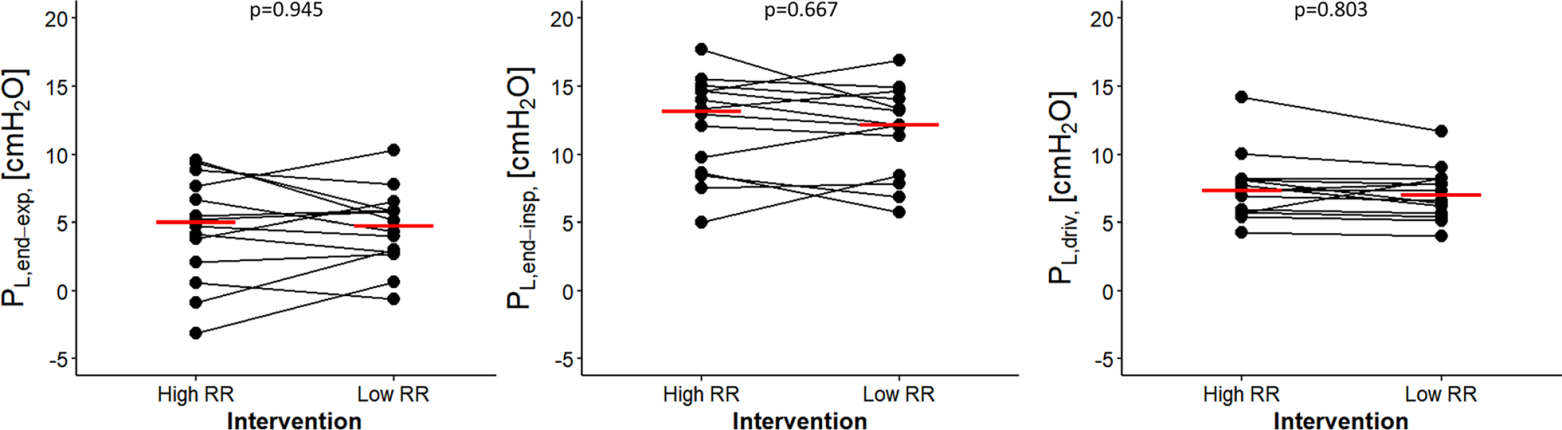
Transpulmonary pressures according to high or low respiratory rate. No differences were observed in transpulmonary pressures between RR interventions. Each point represents one patient

No differences were observed in respiratory system compliance, driving pressure, plateau airway pressure and mean airway pressure between high and low RR (Table 2). Peak airway pressure and airway flow were lower during ventilation using a lower RR. An esophageal catheter was placed in 14 patients. No differences were observed in transpulmonary and esophageal pressures between the two groups (Fig. 4). The specific values and representative tracings are shown in supplementary Table S4, and supplementary Figure S7, respectively.

## Discussion

The findings of this cross-over study in patients with moderate to severe ARDS due to COVID-19 receiving lung-protective invasive ventilation can be summarized as follows: (1) a sustained reduction in RR for 12 h had no impact on lung injury biomarkers, hemodynamics, or respiratory mechanics; (2) these results occurred despite significant reductions in minute ventilation and MP; and (3) it seems feasible to apply low RR ventilation while maintaining lung-protective parameters and remaining within target values for PaCO_2_ and pH. Finally, this pilot study may inform the design of future investigations on MP-targeted ventilation strategies.

To our knowledge, this is one of the first clinical studies specifically designed to assess the effects of reducing RR. We successfully reduced the RR and minute ventilation by one-third. This induced hypercapnia; however, PaCO₂ and pH levels remained within safe limits. The concept of permissive hypercapnia, described more than 30 years ago, is widely accepted as a consequence of lung-protective strategies [26]. Although somewhat controversial, recent studies in patients with sepsis and ARDS have found no evidence of either benefit or harm from hypercapnia [27, 28].

Mechanical power has gained increasing attention as a unifying concept that quantifies the energy delivered to the lungs per unit of time and may ultimately be responsible for VILI [29]. Observational studies in both ARDS patients and patients without ARDS have demonstrated a relationship between mechanical power and adverse outcomes [3, 5, 29]. In this study, the sustained reduction in RR led to a significant decrease in MP, bringing MP values below thresholds for harm observed in previous observational clinical studies [4]. Our results indicate that reducing RR is clinically feasible and could provide a foundation for investigating RR as a potential lung-protective strategy [30].

We did not observe changes in plasma levels of IL-6 nor in other lung injury biomarkers when lowering RR. Although IL-6 has been proven to be a marker of VILI, it may not fully represent the entire pathophysiology of VILI. Baseline values for IL-6 were lower than expected from studies in non-COVID-19 ARDS but were consistent with recent studies that have evaluated the inflammatory response in COVID-19 [31, 32]. Other factors, including time exposure and the applied protective ventilation strategy, may also explain these results. Both high and low RR strategies were implemented under strict protective invasive ventilation protocols, which included low Vt, airway driving and plateau pressures, neuromuscular blockade agents, and prone positioning in almost 80% of patients. In that sense, the MP observed in this study may not have been sufficient to induce a detectable change in soluble inflammatory markers or render the patient susceptible to VILI.

Regarding time exposure, a previous study observed changes in plasma levels of IL-6 and other cytokines within an hour of switching from protective to injurious large tidal volume ventilation [21]. Thus, we assumed that a 12-h period might be sufficient to test the protective effect of decreasing RR. However, longer exposure durations may be required to detect changes in biomarkers or clinical manifestations of VILI under a low tidal volume strategy. Future studies specifically designed to evaluate temporal changes in biomarkers might be also helpful in better understanding these results.

Adjusting the RR can affect hemodynamics through various mechanisms. High RR may shorten expiratory times, leading to auto-PEEP and dynamic hyperinflation [33, 34], or may induce hypocapnia [35, 36]. Conversely, low RRs may induce hypercapnia and pulmonary arterial vasoconstriction. Consequently, changes in RR may result in hemodynamic compromise, including increased pulmonary vascular resistance, decreased cardiac output as well as acute core pulmonale [37]. We observed no changes in hemodynamics or in right ventricular function, most probably because of the absence of auto-PEEP and no differences in mean airway pressures. The absence of auto-PEEP in the present study may be because the study protocol established a maximum RR of 32 breaths/min. The fact that most patients were in prone position and ventilated with moderate PEEP levels and rather low driving pressures may have also decreased the risk of right ventricular dysfunction [38].

Changes in RR have been shown to produce variable impacts on respiratory mechanics [10]. These effects may result from auto-PEEP, or because of modifications of inspiratory or expiratory times and their impact on tidal recruitment or time-dependent collapse [39, 40]. In this study, we observed no impact of RR on respiratory system compliance or in airway pressures. Furthermore, no changes were observed in transpulmonary pressures. The contrast between our results and previous studies [34], may be explained by the absence of auto-PEEP and the maintenance of a fixed inspiratory to expiratory ratio when modifying RR. Time-dependent collapse has been described in experimental models of alveolar instability [39], but it is likely less pronounced in clinical ARDS.

This study has strengths and limitations. It is among the first clinical studies specifically designed to evaluate the effects of reducing respiratory rate RR and, consequently, MP. In addition, it includes a subgroup of patients with advanced respiratory and hemodynamic monitoring, providing valuable data. Although we could not prove our hypothesis, we demonstrated that reducing RR is feasible, which could serve as a foundation for future clinical studies targeting RR and MP.

Regarding limitations, the present study included a relatively small sample size. This was estimated based on prior studies comparing injurious versus protective ventilation strategies without considering RR. As a result, the effect size assumption of a 20% mean difference in biomarker levels might be likely overestimated, potentially leading to an underestimation of the required sample size.

All patients included had COVID-19-related ARDS and monitoring of esophageal pressures, transthoracic echocardiography, and PiCCO measurements were included only in a subset of the population due to pandemic-related constraints. While it seems unlikely that the results would differ in patients with non-COVID ARDS [41], it would be valuable to confirm these findings in a more diverse cohort of ARDS patients in future studies.

In addition, the primary outcome in this study was IL-6. Although IL-6 has been proven to be a marker of VILI, it may not fully represent the entire pathophysiology of VILI. To overcome this potential limitation, we analyzed a wide range of relevant plasma biomarkers of lung injury. Importantly, several factors may have limited our ability to detect significant differences in biomarker levels, including the relatively short 12-h observation period and the fact that cytokine analysis was conducted using plasma samples only, without assessment of BAL concentrations, which may have influenced the findings and contributed to differing results. Furthermore, although we observed clinically relevant differences in median RR values—20 and 30 breaths per minute, respectively—this difference may not have been large enough to elicit detectable changes in cytokine levels within an overall lung-protective ventilation strategy in this population.

Finally, the study was restricted to the first 48 h of mechanical ventilation when elastance was relatively preserved and patients were under deep sedation and controlled mechanical ventilation. Therefore, it is important to interpret these results cautiously, particularly when applying them to patients with late-stage ARDS, or patients with assisted ventilation, in whom RR may have a different impact on VILI.

## Conclusion

In patients with moderate to severe COVID-19-related ARDS during the first 48 h of lung-protective mechanical ventilation, a sustained 12-h reduction in respiratory rate decreased MP but had no impact on lung injury biomarkers. The decrease in RR was feasible, maintaining PaCO₂ and pH within safe limits, and had no effect on hemodynamics or respiratory mechanics.

## Supplementary Information

Supplementary material 1.

## Data Availability

The data sets used and/or analyzed during the current study are available from the corresponding author on reasonable request.
