# Supplementary material for: Effect of decreasing respiratory rate on the mechanical power of ventilation and lung injury biomarkers: a randomized cross-over clinical study in COVID-19 ARDS patients
Source: Intensive Care Med Exp. 2025 Jul 9;13:69. doi: 10.1186/s40635-025-00782-4 (PMC12240881; doi:10.1186/s40635-025-00782-4)
Supplement: Supplementary file 1 — Supplementary material 1. [file 40635_2025_782_MOESM1_ESM.docx]

**SUPPLEMENTARY MATERIAL**

**Effect of decreasing respiratory rate on the mechanical power of ventilation and plasma IL-6 levels – a randomized crossover clinical study in COVID-19 ARDS patients**

L. Felipe Damiani, PhD^1,2^, Roque Basoalto, MSc^1,4^, Vanessa Oviedo, RN^1^, Leyla Alegria RN, MSc^1^, Dagoberto Soto,PhD^1^, , M. Consuelo Bachmann, PT^1^, Yorschua Jalil, MSc^1^, Cesar Santis, MD^1^, David Carpio, MD^1^, Rodrigo Ulloa, MD^1^, Daniel Valenzuela, MD^1^, Magdalena Vera, MD^1^, Marcus J. Schultz, Jaime Retamal, MD, PhD^1^, Alejandro Bruhn, MD, PhD^1^,and Guillermo Bugedo, MD^1*^

**STUDY POPULATION**

***Inclusion Criteria:***

- Patients intubated and under mechanical ventilation with acute respiratory distress syndrome less than 48 hours.
- Acute onset (less than 1 week)
- Chest-X-ray: bilateral infiltrates
- Absence of heart failure or hydrostatic pulmonary edema
- Oxygenation disorder: PaO2/FiO2 ratio <200, with PEEP ≥5 cmH2O*

* For patients in prone position at baseline, the last PaO2/FiO2 ratio in supine (before prone) was considered.

***Exclusion Criteria:***

- Age <18 years
- Previous chronic respiratory disease (chronic obstructive lung disease, asthma, interstitial lung disease, pulmonary fibrosis, chronic bronchiectasis)
- Hypercapnic respiratory failure, defined as PaCO2 >60 mmHg or pH<7.25 despite a RR >30.
- Concomitant severe metabolic acidosis: pH<7.20
- Catastrophic respiratory failure, defined as PaO2/FiO2 ratio <80, despite optimization of ventilatory parameters, or need for ECMO.
- Contraindication to hypercapnia, such as intracranial hypertension or acute coronary syndrome
- Use of vasoconstrictor drugs in increasing doses in the last 2 hours (≥0.5 μg/kg/min of noradrenaline) or average blood pressure <65mmHg
- Pneumothorax or subcutaneous emphysema not drained.
- Pregnancy
- Presence of mental or intellectual disability prior to hospitalization
- Early limitation of therapeutic effort

**LUNG INJURY BIOMARKERS**

***Plasma processing:*** Blood collected in green-top (sodium heparin) blood collection tubes (BD Vacutainer) was subjected to centrifugation at 1000xg for 10 min, and the plasma was aliquoted into 1.5 ml microtubes, and frozen at −80 °C until determination. Prior to cytokine assays, frozen plasma samples were thawed completely and thoroughly mixed.

***Multiplexed cytokine assay:*** The luminex R&D kit for quantification of 7 human cytokines and chemokines (IL-6, IL-8, TNF- α receptor [TNF-RI]), markers of epithelial and endothelial lung injury including surfactant protein (SP-D), soluble receptor of advanced glycation end-products (RAGE), angiopoietin-2 and early pro-fibrotic activity (TGF-β)) was customized and purchased from R&D System (https://www.rndsystems.com/luminex/analytes). Assays were performed according to the manufacturer’s instructions. Briefly, after plates were pre-wet, 50 μl of precombined beads was added and washed twice. Plasma samples (25 μl) were diluted 1:1 with serum matrix and added to the plate. The plate was shaken for 30 seconds at 500 rpm and then incubated for one hour on a plate shaker at 700 rpm at room temperature. Plates were washed twice, 25μl of detection antibody was added per well, and plates were incubated for one hour on a plate shaker. 50μl of strepatavidin-PE conjugate was added to each well, and the plate was shaken at 500 rpm for 30 minutes at room temperature. Finally, plates were washed three times and 150 μl of sheath fluid were added to each well. Plates were read using a Luminex machine. Data was analyzed according to the Luminex Instrument instructions. A standard curve for each cytokine was generated by mixing known concentrations of recombinant human cytokines.

**TRANSTHORACIC ECHOCARDIOGRAPHY**

Transthoracic echocardiography examinations (Mindray Bio-Medical Electronics Co., Shenzhen. China) were performed for left and right ventricular assessment (1,2). Two, four, and five chambers apical views were used to determine left ventricular ejection fraction (LVEF), and aortic velocity time integral (VTI). Stroke volume (SV) was calculated as VTI × Aortic area. End-diastolic right ventricle/left ventricle (RV/LV) area ratio was measured in four apical views. Peak velocity tricuspid regurgitation (TRmax) with continuous wave Doppler were measured, and pulmonary artery systolic pressure (PASP) estimated. Cor pulmonale was defined as a dilated right ventricle (end-diastolic RV/LV area ratio > 0.6) associated with septal dyskinesia on the short-axis view.

**References**

1. Ugalde D, Medel JN, Mercado P, et al. Critical care echocardiography in prone position patients during COVID-19 pandemic: a feasibility study. J Ultrasound. 2022;25(4):855-859.
2. Valenzuela ED, Mercado P, Pairumani R, et al. Cardiac function in critically ill patients with severe COVID: A prospective cross-sectional study in mechanically ventilated patients. J Crit Care. 2022;72:154166.

**SUPPLMENTARY TABLES**

| **Table S1. Plasma levels biomarkers of inflammation and lung injury at baseline, low and high respiratory rate** | | | | |
| --- | --- | --- | --- | --- |
|  | **Baseline (n=32)** | **Low RR (n=32)** | **High RR (n=32)** | **p-value*** |
| **Inflammation** |  |  |  |  |
| Interleukin-6, pg/mL | 13 [7-38] | 14 [7-22] | 11 [7-22] | 0.506 |
| Interleukin-8, pg/mL | 41 [22-61] | 37 [19-51] | 35 [23-46] | 0.674 |
| TNF-R1, pg/mL | 2,529 [1,947-3,485] | 2,714 [2,066-3,020] | 2,447 [1,988-2,997] | 0.591 |
| **Lung epithelial injury** |  |  |  |  |
| SP-D, pg/mL | 14,440 [6,287-29,134] | 14,370 [8,131-27,540] | 14,408 [7,539-29,429] | 0.984 |
| RAGE, pg/mL | 4,845 [2,080-7,435] | 2,880 [1,558-5,858] | 2,930 [1,430-5,244] | 0.846 |
| **Endothelial injury** |  |  |  |  |
| Angiopoietin-2, pg/mL | 2,775 [1,637-5,623] | 2,507 [1,710-5,610] | 2,618 [1,646-5,756] | 0.918 |
| **Pro-fibrotic activity** |  |  |  |  |
| TGF-ß, pg/mL | 3,185 [1,121-5,503] | 3,482 [1,469-32,401] | 2,998 [1,029-5,712] | 0.641 |
| Results are presented as median [p25%-p75%]. Abbreviations: SP-D, surfactant protein D; RAGE, Receptor for advanced glycation end products; TGF-ß, transforming growth factor-ß. * Comparison between high and low RR using Wilcoxon signed-rank test. | | | | |

| **Table S2. Plasma levels biomarkers of inflammation and lung injury grouped by study period** | | | | |
| --- | --- | --- | --- | --- |
|  | **Baseline (n=32)** | **Period 1 (12h; n=32)** | **Period 2 (24h; n=32)** | **p-value*** |
| **Inflammation** |  |  |  |  |
| Interleukin-6, pg/mL | 13 [7-38] | 11 [7-20] | 14 [7-23] | 0.677 |
| Interleukin-8, pg/mL | 41 [22-61] | 35 [18-49] | 37 [24-48] | 0.561 |
| TNF-R1, pg/mL | 2,529 [1,947-3,485] | 2,585 [2,079-2,877] | 2,649 [1,988-3,177] | 0.667 |
| **Lung epithelial injury** |  |  |  |  |
| SP-D, pg/mL | 14,440 [6,287-29,134] | 14,408 [7,539-27,540] | 14,498 [8,247-29,471] | 0.867 |
| RAGE, pg/mL | 4,845 [2,080-7,435] | 3,709 [1,571-6,660] | 2,507 [1,459-4,536] | 0.483 |
| **Endothelial injury** |  |  |  |  |
| Angiopoietin-2, pg/mL | 2,775 [1,637-5,623] | 2,425 [1,670-5,498] | 2,862 [1,694-6,077] | 0.769 |
| **Pro-fibrotic activity** |  |  |  |  |
| TGF-ß, pg/mL | 3,185 [1,121-5,503] | 2,819 [1,099-5,712] | 3,691 [1,117-32,759] | 0.835 |
| Results are presented as median [p25%-p75%]. Abbreviations: SP-D, surfactant protein D; RAGE, Receptor for advanced glycation end products; TGF-ß, transforming growth factor-ß. * Comparison between period 1 and period 2 using Wilcoxon signed-rank test. | | | | |

| **Table S3. Hemodynamic and cardiac function according to high or low respiratory rate** | | | |
| --- | --- | --- | --- |
| **Variable** | **Low RR (n=12)** | **High RR (n=12)** | **p-value** |
| **Ventilatory parameters** |  |  |  |
| Respiratory Rate, breaths/min | 20 [18-22] | 30 [28-32] | 0.001 |
| PaO2/FiO2 ratio | 177 [128-217] | 193 [133-203] | 0.781 |
| Tidal volume, ml | 403 [366-442] | 402 [365-437] | 0.84 |
| Peak airway pressure, cmH2O | 24 [22-28] | 27 [25-30] | 0.039 |
| Plateau pressure, cmH2O | 22 [20-27] | 23 [20-28] | 0.485 |
| Mean airway pressure, cmH2O | 15 [13-19] | 15 [14-20] | 0.599 |
| PEEP, cmH2O | 11 [10-16] | 11 [10-16] | 0.999 |
| Inspiratory oxygen fraction | 0.53 [0.36-0.6] | 0.43 [0.40-0.57] | 0.539 |
| Inspiratory time, sec | 1 [0.91-1.13] | 0.67 [0.60-0.73] | 0.001 |
| Inspiratory:Expiratory ratio | 0.6 [0.5-0.7] | 0.59 [0.51-0.70] | 0.755 |
| **Acid base state** |  |  |  |
| pH | 7.34 [7.32-7.38] | 7.46 [7.43-7.49] | 0.001 |
| PCO2, mmHg | 55 [53-60] | 40 [36-43] | 0.014 |
| PO2, mmHg | 82 [76-97] | 81 [73-90] | 0.44 |
| Bicarbonate, mEq/L | 30 [27-34] | 27 [25-30] | 0.148 |
| **Macro-hemodynamic** |  |  |  |
| SBP, mmHg | 108 [99-122] | 116 [104-121] | 0.623 |
| DBP, mmHg | 66 [58-74] | 70 [62-73] | 0.563 |
| MAP, mmHg | 78 [74-93]] | 89 [78-92] | 0.544 |
| NE, mcg/kg/min | 0.0 [0.00-0.01] | 0.0 [0.0-0.02] | 0.89 |
| HR, beats/min | 69 [56-74] | 56 [50-64] | 0.133 |
| CVP, mmHg | 14 [10-16] | 10 [7-14] | 0.097 |
| **Tissue perfusion parameters** |  |  |  |
| Capillary Refill Time, sec | 2.0 [1.0-3.0] | 1.5 [1.0-2.0] | 0.162 |
| Lactate, mmol/L | 1.3 [1.0-1.8] | 1.7 [1.6-1.9] | 0.056 |
| **CO and LV function** |  |  |  |
| Cardiac output, L/min (PiCCO) | 6.8 [4.7-8.3] | 5.4 [4.9-7.1] | 0.481 |
| EVLW | 930 [418-1300] | 1270 [367-1524] | 0.596 |
| LV Ejection Fraction, % | 58 [44-67] | 61 [50-66] | 0.526 |
| LVOT VTI, cm | 23 [15-24] | 20 [15-23] | 0.895 |
| Stroke Volume, ml | 72 [59-87] | 76 [55-91] | 0.825 |
| MAPSE, mm | 13 [12-17] | 15 [14-18] | 0.326 |
| Mitral TDI s’ wave, cm/sec | 10.5 [8.9 13.3] | 11.9 [9.7-16.5] | 0.29 |
| **Right ventricle function** |  |  |  |
| TAPSE, mm | 22.4 [19.7-26.2] | 22.4 [20.5-26.4] | 0.775 |
| Tricuspid TDI s’ wave, cm/sec | 14.3 [11.1-17.9] | 15.0 [10.6-18.5] | 0.848 |
| Right end diastolic area, cm^2^ | 17 [14-23] | 17 [13-23] | 0.999 |
| Left end diastolic area, cm^2^ | 29 [26-38] | 30 [23-35] | 0.859 |
| RVEDA/LVEDA ratio | 0.61 [0.51-0.80] | 0.59 [0.55-0.75] | 0.859 |
| SPAP, mmHg | 36 [24-32] | 33 [26-37] | 0.317 |
| **Diastolic function** |  |  |  |
| Doppler Trans-mitral E wave, cm/sec | 72 [51-76] | 64 [55-74] | 0.778 |
| Doppler Trans-mitral A wave, cm/sec | 53 [39-59] | 61 [49-68] | 0.205 |
| Mitral TDI e’ wave, cm/sec | 11.0 [8.3-12.3] | 9.8 [8.5-12.6] | 0.677 |
| E/e’ ratio | 6.4 [4.7-7.2] | 6.7 [4.7-8.3] | 0.87 |
| **Inferior vena cava (IVC)** |  |  |  |
| Maximum IVC diameter, mm | 21.5 [20.0-22.0] | 20.5 [17.4-22.5] | 0.606 |
| Minimum IVC diameter, mm | 20.0 [19.7-21.0] | 19.5 [17.3-21.4] | 0.897 |
| Continuous data were expressed as median [25th–75th percentiles]. SBP = systolic blood pressure; DBP = diastolic blood pressure; MAP = median arterial pressure; NE = norepinephrine; HR = heart rate; CVP = central venous pressure; LVEDV = left ventricular end diastolic volume; LVESV = left ventricular end systolic volume; MAPSE = mitral annular plane systolic excursion; LVOT = left ventricular outflow tract; VTI= velocity time integral; TAPSE = tricuspid annular plane systolic excursion; TDI = tissue Doppler imaging; RVEDA/LVEDA ratio = right ventricle end diastolic area/left ventricle end diastolic area ratio; ACP = acute cor pulmonale; E/A ratio = E wave/A wave ratio; E/e’ ratio = E wave/tissue Doppler image e’ wave ratio; IVC = inferior vena cava. | | | |

| **Table S4. Esophageal, airway and transpulmonary pressure with high and low respiratory rate** | | | |
| --- | --- | --- | --- |
| **Variable** | **High RR (n=14)** | **Low RR (n=14)** | **p-value*** |
| P_es,end-exp_ (cmH_2_O) | 7.8 [5.8-10.3] | 8.3 [5.5-9.7] | 0.963 |
| P_es,end-insp_ (cmH_2_O) | 10.8 [8.3-13.6] | 11.6 [8.2-12.7] | 0.982 |
| P_es,driv_ (cmH_2_O) | 2.4 [1.8-3.2] | 3.1 [1.9-3.4] | 0.669 |
| P_aw,end-exp_ (cmH_2_O) | 12.2 [10.5-15.4] | 11.9 [10.6-14.2] | 0.668 |
| P_aw,end-insp_ (cmH_2_O) | 22.8 [19.9-27.1] | 22.5 [19-25.6] | 0.713 |
| P_aw_,_driv_ (cmH_2_O) | 10.1 [8.4-11.7] | 9.8 [9.1-10.7] | 0.667 |
| P_L,end-exp_ (cmH_2_O) | 5 [2.5-7.4] | 4.7 [2.9-5.9] | 0.945 |
| P_L,end-insp_ (cmH_2_O) | 13.1 [8.9-14.6] | 12.2 [9.2-13.9] | 0.667 |
| P_L,driv_ (cmH_2_O) | 7.36 [5.8-8.1] | 6.9 [5.8-8.1] | 0.803 |
| Data are presented as median [p25%-p75%]. Abbreviations. Pes,end-insp: Esophageal pressure measured at end inspiration occlusion; Pes,end-exp: Esophageal pressure measured at end expiration occlusion; Pes,driv: Driving esophageal pressure; Paw,end-insp: Airway pressure measured at end inspiration occlusion; Paw,end-exp: Airway pressure measured at end expiration occlusion; Paw,driv: Driving airway pressure; PL,end-insp: Transpulmonary pressure measured at end inspiration occlusion; PL,end-exp: Transpulmonary pressure measured at end expiratory occlusion; PL,driv: Lung driving pressure (i.e., difference between PL,end-insp and PL,end-exp). * p-value for High RR v/s Low RR comparison using Wilcoxon signed-rank test. | | | |

**SUPPLEMENTARY FIGURES**

**Figure S1. Study protocol.**

**
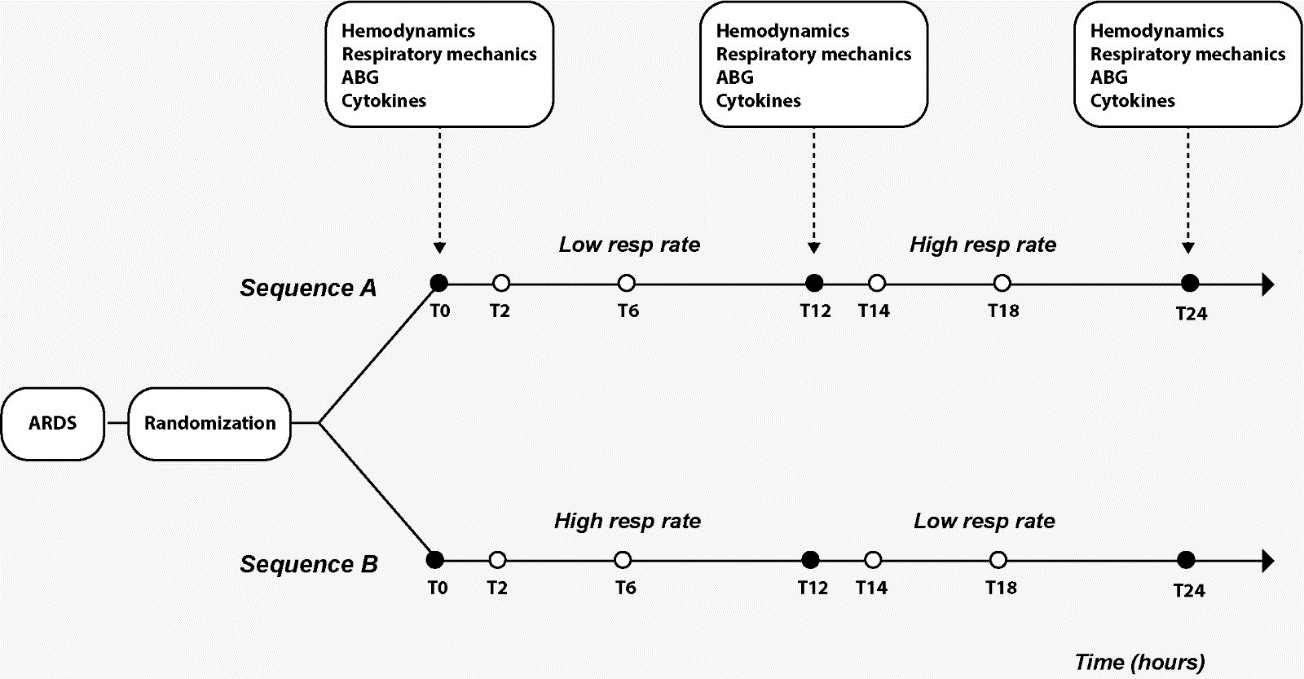
**

**Figure S2. Algorithm for setting target respiratory rate.**





Figure S2. High and low respiratory rate were set considering baseline respiratory rate, pH, and PaCO2 parameters. The first step is to locate pH (a) and PaCO2 (b) values obtained at baseline in the corresponding RR. Target frequencies were defined when both pH and PaCO2 are maintained within the safe limits and a minimum of 8-point difference in RR is present.

**Figure S3. Evolution of respiratory rate for each patient**

**
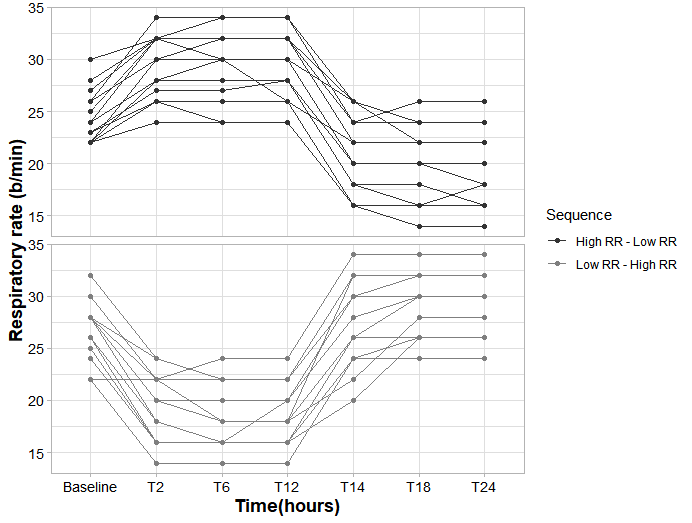
**

**Figure S4. Plasma levels of IL-6 according to ARDS severity, respiratory system compliance and Baseline level of IL-6**

**
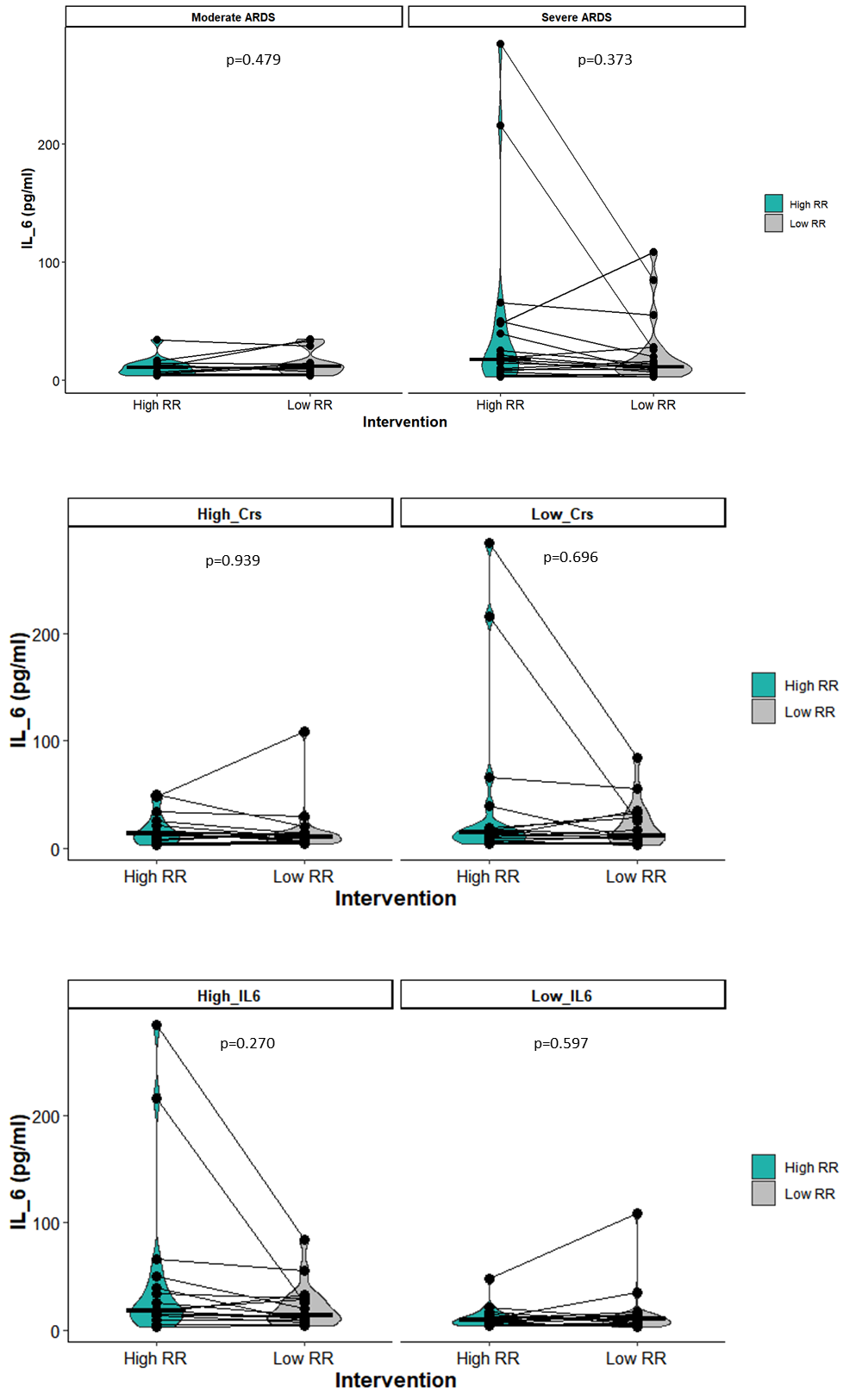
**

**Figure S5. Plasma levels of IL-8, TNF- α receptor, and surfactant protein-D according to ARDS severity**

**
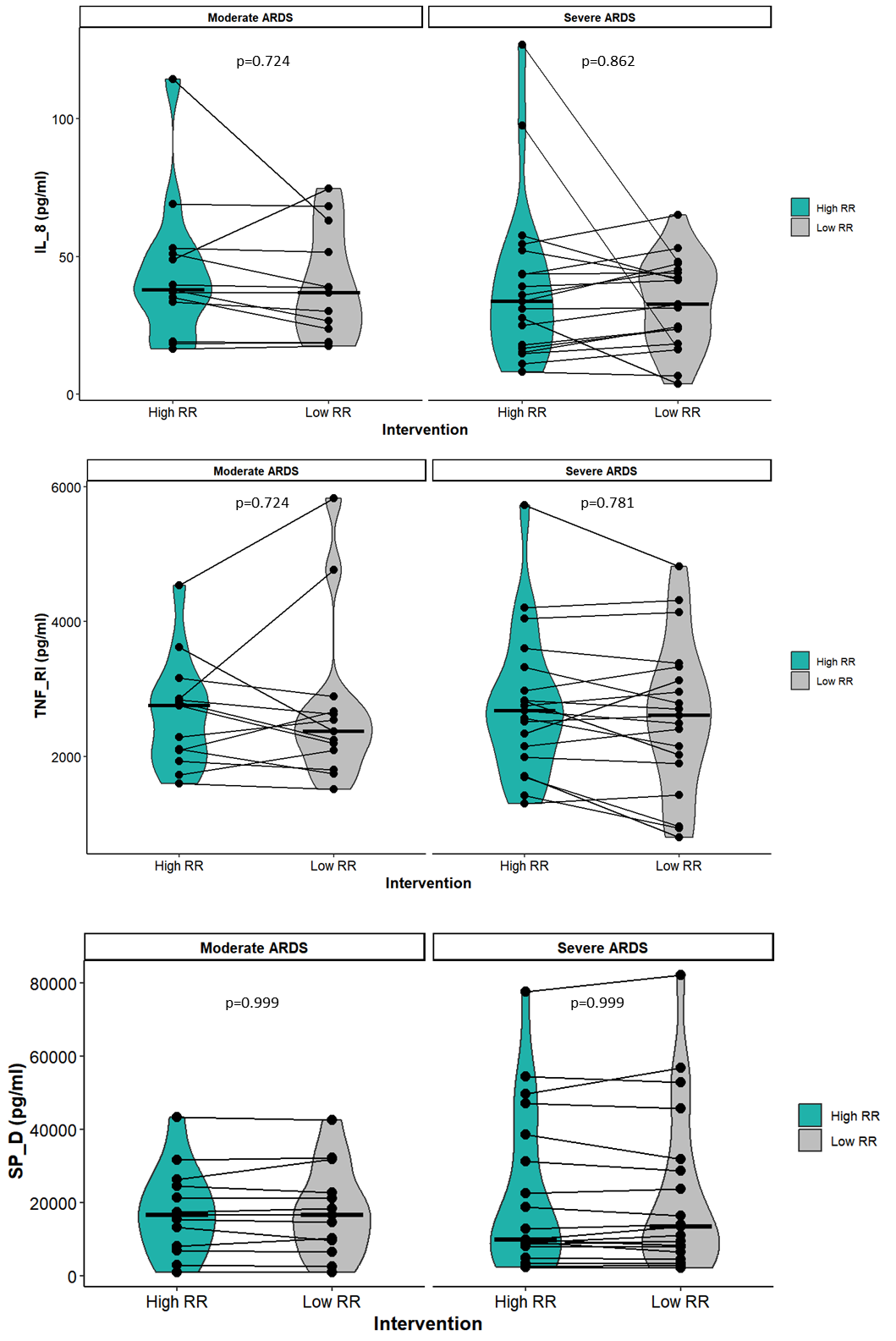
**

**Figure S6. Plasma levels of RAGE, angiopoietin-2, and TGF-β according to ARDS severity**

**
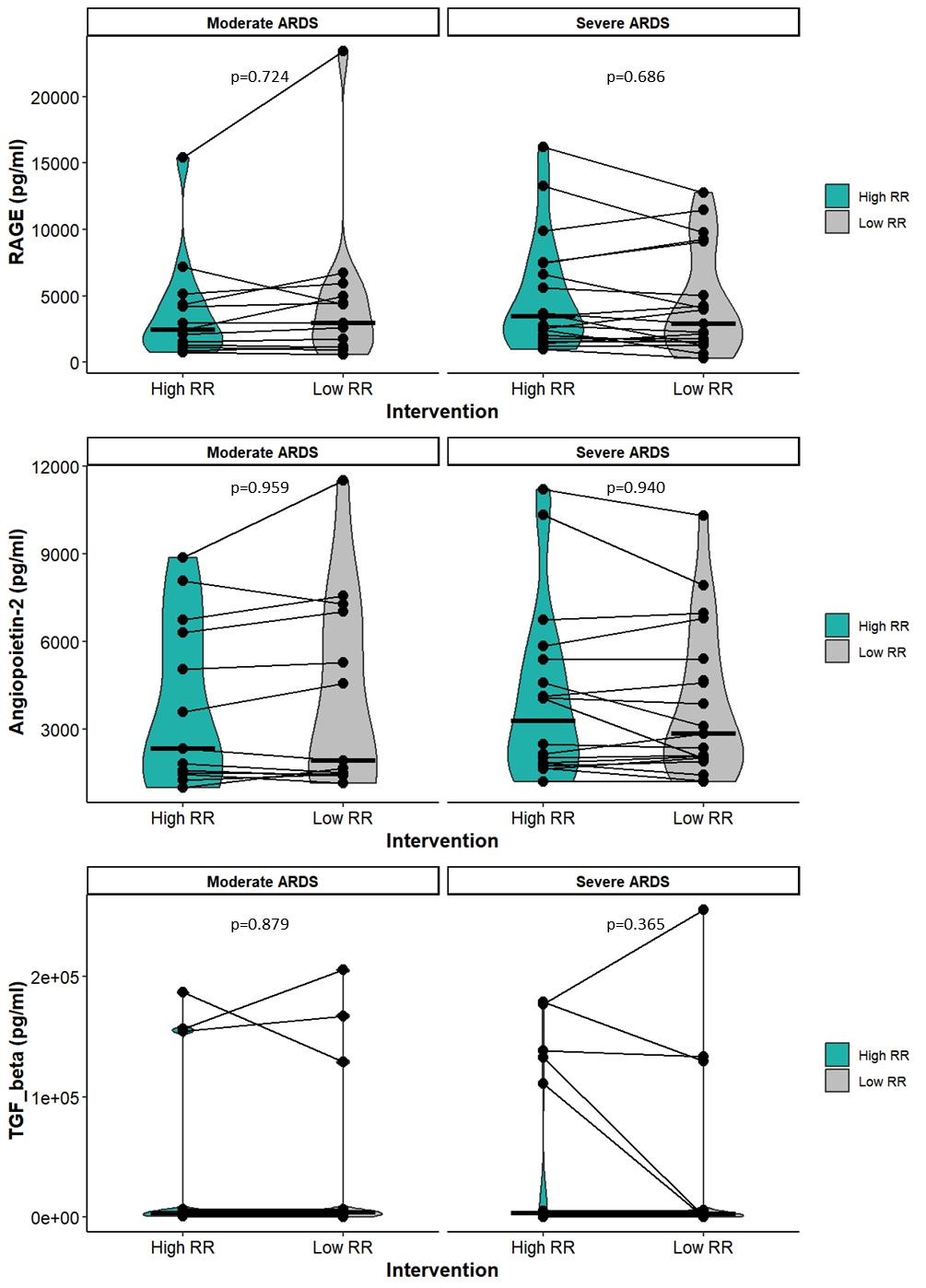
**

**Figure S7. Example of high vs low respiratory rate in one patient**


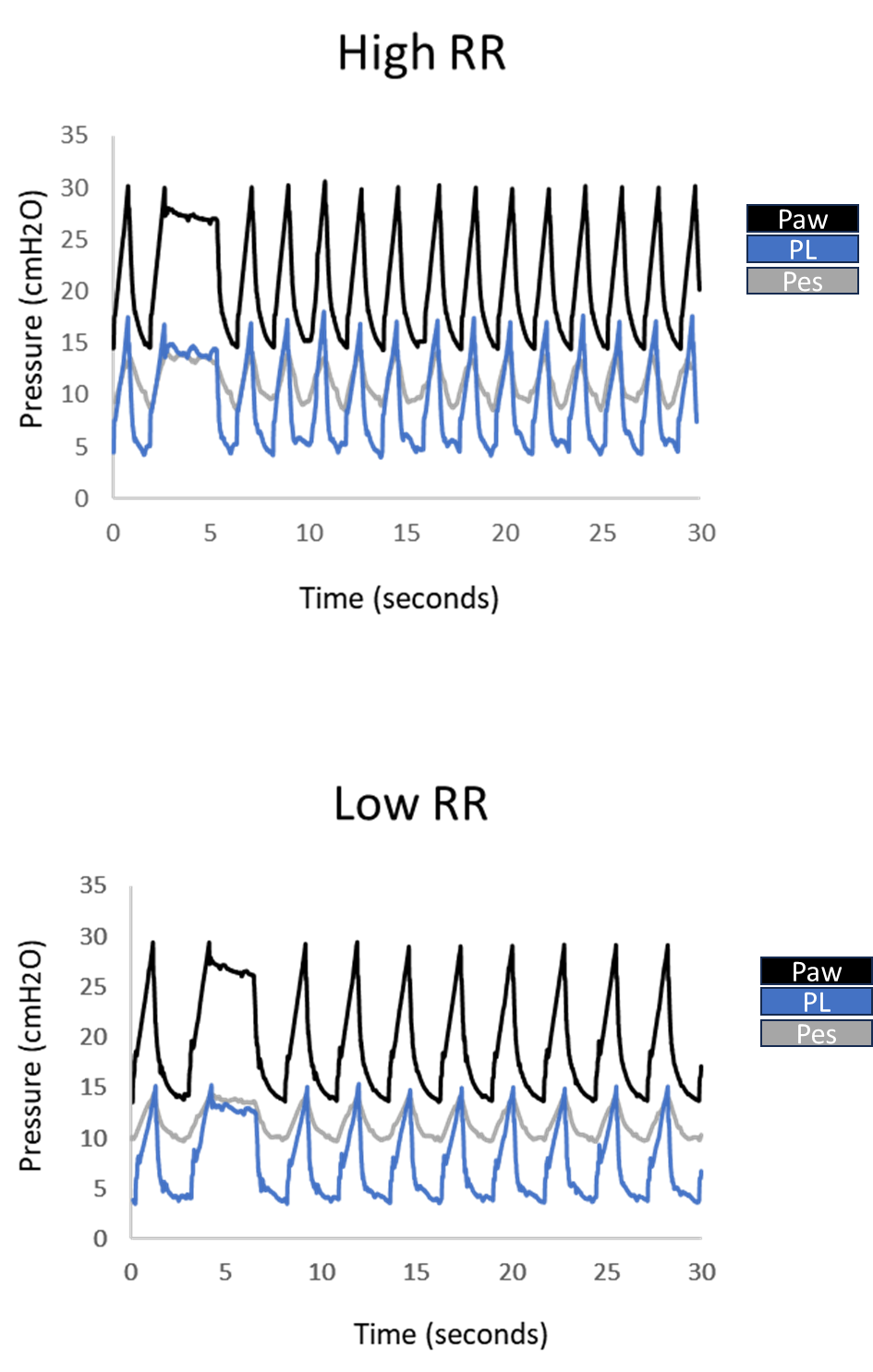


Fig S5. Representative tracings for one patient shoeing airway (black), esophageal (gray) and transpulmonary pressure (blue) during high (30 breaths/min) and low respiratory rate (20/breaths min) strategy. Paw: airway pressure; PL: transpulmonary pressure; Pes: esophageal pressure.
